# Micronutrient deficiencies after pancreatico‐duodenectomy: A narrative review of the literature and recommendations for clinical practice

**DOI:** 10.1002/ncp.70007

**Published:** 2025-07-30

**Authors:** Mary E. Phillips, Callum Livingstone, Adam E. Frampton, Kathryn H. Hart

**Affiliations:** ^1^ Faculty of Health and Medical Sciences, University of Surrey Guildford UK; ^2^ Department of Nutrition and Dietetics Royal Surrey Hospital Guildford UK; ^3^ Department of Clinical Biochemistry Royal Surrey Hospital Guildford UK; ^4^ Department of HPB Surgery Royal Surrey Hospital Guildford UK; ^5^ Department of Oncology University of Surrey Guildford UK

**Keywords:** clinical practice, deficiency, depletion, micronutrients, pancreatico‐duodenectomy

## Abstract

Micronutrient uptake is impaired after pancreatico‐duodenectomy (PD) because of malabsorption, reduced absorptive capacity, and poor oral intake. Biochemical depletion is reported in cohort studies, but deficiency states are predominantly reported in case reports, making it difficult to assess occurrence rates. Although national guidelines recommend monitoring of micronutrients, there are no guidelines on what this should consist of. We undertook a narrative review to explore the incidence of deficiency and make recommendations for clinical management using data from randomized controlled trials, cohort studies, and case reports. We established that iron, zinc, and vitamin D deficiencies are common. Fat‐soluble vitamin deficiencies are rare and occurred in patients who discontinued pancreatic enzymes but are otherwise nutritionally well, whereas trace element and B vitamin deficiencies occurred as part of a more generalized malnutrition state. We make recommendations for identification and treatment of micronutrient deficiencies and recommend routine assessment of iron, zinc, and vitamin D status and bone density in all patients who have undergone curative treatment and take pancreatic enzyme replacement therapy (PERT) or have benign disease. In those with malnutrition or not taking PERT, additional screening for vitamins A, E, and K; selenium; copper; and magnesium should be undertaken. A pragmatic approach should be taken for those with limited long‐term survival, with a focus on iron status, as this will impact quality of life.

## INTRODUCTION

National Institute for Health and Care Excellence (NICE) guidelines for nutrition support highlight the need for vitamin and mineral biochemical assessment in patients receiving artificial nutrition,^1^ and recommendations exist for patients who have had bariatric surgery.^1^ We have previously suggested micronutrient deficiency screening after pancreatico‐duodenectomy (PD),^2^ but these recommendations were based on expert opinion given the paucity of data at the time.

Micronutrient status has been analyzed in several observational cohort studies in patients who have undergone PD^3^, ^4^, ^5^, ^6^, ^7^, ^8^, ^9^, ^10^ or total pancreatectomy (TP).^11^ Resection of the duodenum and use of the 30–40 cm of the proximal jejunum within the reconstruction reduces the absorptive capacity within the proximal small bowel and may be associated with poor nutrient uptake. Furthermore, maldigestion occurs secondary to pancreatic exocrine insufficiency (PEI), which may result in osmotic diarrhea, further reducing absorption. Fat maldigestion predisposes patients to poor uptake of fat‐soluble vitamins,^12^ and this is often progressive after PD because the pancreatic remnant can atrophy with time.^13^

PD is carried out predominantly for pancreatic ductal adenocarcinoma (PDAC), which has a poor 5‐year survival rate.^14^ Other conditions for which PD is indicated have better long‐term survival rates, and thus, most cohort studies include patients with other less aggressive tumors, premalignant conditions, and benign disease.^4^, ^8^, ^11^

The signs and symptoms of deficiency may not be obvious and may require clinical assessment. For instance, osteoporosis is associated with deficiencies in vitamins B_12_, D, and K; copper; zinc; manganese; fluoride; and boron^15^ but will not be apparent unless a bone density scan is undertaken or the patient has a low‐impact fracture. Thus, there is a need to identify nutrient depletion early to try to prevent the clinical consequences of deficiency.

Furthermore, signs of deficiency such as poor wound healing or immune dysfunction are multifactorial,^16^, ^17^ and a nutrition cause may not be identified.

The aim of this paper is to review the incidence of micronutrient deficiency and make practical recommendations for the screening, identification, and management of deficiencies.

## METHODS

This narrative review describes the presentation and management of micronutrient deficiencies after PD, using data from clinical trials, case studies, and clinical guidelines to present recommendations for clinical practice.

Data were sourced from Medline searches including each individual nutrient (eg, “zinc” and “selenium”) and nutrition state (eg, “osteoporosis” and “acrodermatitis enteropathica”) in addition to the collective terms “malnutrition; micronutrients,” “trace elements,” “deficiency,” and “depletion.” Hand searches of references of the literature reviewed, textbooks, and published guidelines within the clinical field were undertaken. All results (randomized controlled trials, observational cohort studies, and case reports, including those published in abstract form only) were included. All case reports were reviewed to ensure cases were appropriate to the cohort being explored. Data not available in English were excluded. Data identified from each micronutrient are presented individually, and general recommendations were made where appropriate.

Micronutrients are defined as “vitamins” (specifically, A, C, D, E, K, and individual B vitamins) and “trace elements” (boron, copper, fluoride, iron, magnesium, selenium, and zinc).^18^ Boron and fluoride were excluded from this paper because we do not have the facilities to assess status in a clinical setting; and calcium—not typically considered a trace element—was included because of the association with bone health, although the management of serum calcium and magnesium depletion requires more acute management than other micronutrients.

Ethical committee review was not required for this literature review–based study.

## RESULTS AND DISCUSSION

The first study to report micronutrient deficiencies after PD was published by Dresler et al. in 1991 and included a cohort of 35 patients who underwent TP over a period of 15 years.^11^ This study reported biochemical micronutrient depletion and significant bone loss in a small number of patients despite supplementation.^11^ It was >20 years later when Armstrong et al. published the next study exploring biochemical micronutrient status.^6^ Subsequent small cohort observational studies have reported varied results over different time frames.^3^, ^5^, ^7^, ^8^, ^9^, ^10^ Most authors combined data from patients from 6 months to >10 years after surgery, with only one paper exploring the onset of deficiencies at different time frames and identifying the same risk of deficiency between year 1 and year 10 after PD.^4^ Three authors excluded those patients with evidence of an acute‐phase response,^3^, ^4^, ^5^ which can adversely affect biochemical assessment,^19^ and dietary intake was not assessed alongside biochemical micronutrient status in any study, but is reported separately as being less nutritionally complete than that of participants' spouses in one small study.^6^

We undertook a large retrospective study and identified that with routine supplementation, most biochemical micronutrient depletions could be prevented^4^; however, levels of zinc, vitamin D, and iron depletion remained consistent with other studies in which patients did not receive supplementation (Table 1). Despite high levels of biochemical depletion, we only observed a small number of clinical deficiency states, but this could be explained by the routine assessment of micronutrient status and correction of biochemical depletion before clinical signs became apparent, which has been standard practice in our institution for >20 years.

**Table 1:** Incidence of biochemical depletion of micronutrients in patients who have undergone pancreatico‐duodenectomy.

	Time from surgery, months	Inflammation	Supplementation			*N*	Participants with depletion, %					
			Calcium	Vitamin D	Multivitamin and mineral		Vitamin A	Vitamin D	Vitamin E	Selenium	Zinc	Iron
Dresler et al^11^	Mean ± SD, 39 ± 6	N/S	✓	✓	✓	<35	>11	>11	>3	N/S	>3	N/S
Armstrong et al^6^	Median (IQR), 23 (8–84)	Excluded	X	X	X	37	N/S	24	N/S	56	N/S	N/S
Latenstein et al^9^	Median (IQR), 13 (7–28)	N/S	X	X	X	85	21	40	3	N/S	4	34 female; 60 male
Tabriz et al^7^	12	N/S	X	X	X	47	N/S	>50	0	N/S	0	19
Murphy et al^5^	Preoperative	Excluded	X	X	X	28	0	57	0	24	83	N/S
Percy et al^8^	Up to 150	N/S	X	X	X	58	40 (fat‐soluble vitamin deficiency)			49 (trace element deficiency)		25
Kroon et al^10^	Up to 18	N/S	X	X	X	24	0	47 (12 months); 53 (18 months)	4 (12 months); 6 (18 months)	N/S	N/S	N/S
Phillips et al^4^	Up to 168	Excluded	✓	✓	✓	205	3	46	2	3	44	42

Abbreviation: N/S, not stated.

European guidelines were published in 2022 to try and standardize the terminology used to determine the micronutrient status of patients, defining depletion as “evidence of objective loss of a micronutrient in body fluids or an intake below standard recommendations or biochemical levels below the reference range.”^18^ Deficiency was defined as depletion with the addition of signs and symptoms of deficiency or metabolic effects of inadequacy.^18^

Given this definition, published data lean toward reporting depletion rather than deficiency, with only one study reporting minimal numbers of symptomatic patients.^4^ Biochemical deficiency requires careful interpretation because of the impact of inflammation,^20^ acute illness, and specific medical conditions. For this reason, serum C‐reactive protein (CRP) and albumin levels should be measured with each vitamin and mineral screen.^18^ CRP levels >20 mg/L will impact the interpretation of biochemical nutrient levels, and several nutrients are bound to serum albumin levels.^18^ Advice should be sought from a clinical biochemist in complex cases.

### Vitamin deficiencies

#### Vitamin A

Cases of vitamin A deficiency with clinically proven signs after PD are rare in the literature,^21^, ^22^, ^23^, ^24^, ^25^ and this may be a result of substantial body stores. Vitamin A is stored in the liver, with sufficient stores to prevent deficiency for at least 4 months, even with a complete absence of dietary intake of vitamin A.^26^

There is an incidence of biochemical deficiency of 11%–21% in unsupplemented patients after PD,^6^, ^9^, ^11^ but there are infrequent clinical manifestations, with a literature search specific to PD revealing only four cases,^21^, ^22^, ^25^, ^27^ although further cases were reported in patients with metastatic pancreatic tumors^23^ and pancreatic neuroendocrine tumors (NETs).^24^, ^28^

The first case identified in the literature is from our own institution. A 37‐year‐old woman presented 6 years after a PD for a benign myofibroblastic tumor. She was 33 weeks pregnant with clinical symptoms of night blindness and anemia. She was prescribed pancreatic enzyme replacement therapy (PERT) postoperatively but had discontinued PERT 18 months previously; her serum vitamin A level was 0.2 μmol/L (reference range, 0.92–2.76), which increased to 0.56 μmol/L after infusion of 6600 IU of vitamin A in a combined fat‐soluble vitamin preparation typically used as an additive for parenteral nutrition (PN). She had a recurrence of symptoms 7 days later and required two further infusions. She had an uneventful delivery at 38 weeks' gestation, after which she was discharged with a prescription of PERT and an oral multivitamin and mineral providing 3750 IU of vitamin A per day.^21^

A second case, presented in abstract form only by Appaswamy et al, is that of a 79‐year‐old woman in the UK who presented 4 years after PD for NET and 2 years after disease recurrence (untreated).^25^ She presented with symptoms of night blindness, and eye examination confirmed both rod and cone dysfunction. Her serum vitamin A level was 0.13 μmol/L (reference range, 0.99–1.35 μmol/L), and she was given an intermuscular injection of 100,000 IU of vitamin A. Her symptoms resolved, and serum vitamin A returned to normal after two injections, with no symptom recurrence with regular vitamin A injections. The authors reported her weight to be stable, and she was receiving a regular prescription of PERT on presentation, although doses and adherence were not specified. There is no mention of other fat‐soluble vitamin levels.^25^ A further case occurred in Australia in a 61‐year‐old gentleman who had a PD for intraductal papillary mucinous neoplasm 10 years previously and discontinued his PERT. Tiang et al^22^ reported a presentation of night blindness during a routine diabetes review, accompanied by anemia and dry skin. The patient's serum vitamin A, D, and E levels were all very low (vitamin A < 3 μg/L, vitamin D 18 nmol/L, and vitamin E < 5 μg/L) with bilateral absent rod responses on electroretinography and a reduction in Arden ratios in each eye, consistent with nyctalopia (night blindness). He was treated with fat‐soluble vitamins and PERT, and his ophthalmological assessments repeated at 2 months with significant improvement.^22^

Kontas et al. reported a case occurring in the UK in a 62‐year‐old man 2.5 years after PD and during active treatment for recurrent pancreatic adenocarcinoma. He presented with symptoms of night blindness and poor color perception. His serum vitamin A level was 0.03 μmol/L (reference range, 1.05–2.80 μmol/L). He responded clinically to intramuscular vitamin A injections, and his treatment was maintained on high‐dose supplements. There was no comment on this patient's general nutrition health, but the authors highlighted the improvement in quality of life with treatment.^27^

Thus, vitamin A deficiency is documented several years after PD, with all four reported cases occurring in the UK and Australia, and may be more likely in patients who are not taking PERT. These cases also highlight the risk of vitamin A deficiency in those who are otherwise nutritionally well.^21^, ^25^

#### Vitamin D and bone health

Studies on a mixed cohort of 75 patients with NETs, including pancreatic NET (*n* = 8), and patients who had undergone duodenal resection (*n* = 7) and pancreatic resection (*n* = 20), of whom eight were receiving PERT, found significantly more frequent vitamin D depletion than in controls (*P* < 0.0001), and severe vitamin D depletion was present in 57% of patients with NET. Furthermore, vitamin D depletion was associated with a reduction in progression‐free survival compared with those with vitamin D sufficiency (*P* = 0.014), but there was no correlation with disease‐specific survival (*P* = 0.46).^29^

A large study (*n* = 8080) of patients who had undergone pancreatectomy, compared with propensity‐matched controls, identified a 2.4‐fold increase in pathological fractures (*P* < 0.0001), and despite a higher incidence of vitamin D supplementation (*P* < 0.0001), there was a higher incidence of osteoporosis/osteomalacia and vitamin D depletion (*P* < 0.0001) when assessed ≥3 years after surgery.^30^ This study used computed tomographic (CT) images to quantify bone density in a subgroup of 224 patients and demonstrated a more rapid decline in bone function compared with historical controls (*P* = 0.015).^30^

Vitamin D supplementation is recommended in the UK for the whole population in the winter months at a daily dose of 10 μg (400 IU).^31^ This is well below levels associated with toxicity, and higher doses are often required to correct deficiency. However, this should be undertaken with biochemical monitoring and in response to a proven deficiency, as vitamin D toxicity can cause significant morbidity. Manifestations of vitamin D toxicity include confusion, poor concentration, irritability, fatigue, general weakness, anorexia, constipation, nausea, vomiting, and in severe cases, pancreatitis or coma.^32^ A dosage of 1200 IU was routinely prescribed in one study over many years after PD with no reported toxicity, but the incidence of depletion was reported as 46% despite this level of supplementation.^4^

A case series of 16 cases of vitamin D toxicity collated over a 2‐year period in a single Indian institution demonstrated presenting complaints including nausea and vomiting, altered sensation, constipation, acute kidney injury, weight loss, and pancreatitis. In all cases, vitamin D had been prescribed by a physician, but in only two cases was a low serum vitamin D level the trigger for prescription. The remaining clinical indications were backache (*n* = 5), nonspecific body aches (*n* = 6), and fatigue (*n* = 2).^32^ Prescriptions were all high dose with a median total dose of 3,600,000 IU (range, 2,220,000–6,360,000) with individual doses of 600,000 IU as an intramuscular injection or oral doses of 60,000 IU/day over 1–3 months. Twelve of the 16 patients required an initial hospital admission, and three were readmitted, two with complications from pancreatitis and one with a spinal fracture after a fall associated with behavioral changes secondary to hypercalcemia. Case studies are also reported with over‐the‐counter preparations,^33^ highlighting the need to counsel patients on appropriate doses.

In summary, vitamin D depletion is common and requires a higher dose of vitamin D than recommended for the general population, although further research is required to determine an appropriate dose. There is a higher incidence of fractures compared with controls, which will result in a high healthcare cost; thus, routine supplementation at a dose above standard doses is warranted, with monitoring to ensure toxicity does not occur. Bone density scanning from 12 months after PD should be considered to allow early detection and thus preventative treatment of any bone mineral loss.

#### Vitamin E

There are limited data on vitamin E deficiency. One case study is reported in Japan in a patient with multiple micronutrient deficiencies after surgery for PDAC. The patient presented immobile, with loss of deep sensation in her legs, myopathy, and polyneuropathy, but on correction of her vitamin E levels, she was able to walk again.^34^ In this case, the patient also had deficiencies in vitamins A, B_1_, B_6_, D, and K, some of which are likely to have contributed to her neurological symptoms. Of note, micronutrient supplementation of vitamin E did not occur until 2 years after the onset of symptoms.^34^ A second case was reported in a large cohort study, presenting with muscle weakness responding to intravenous vitamin E supplementation,^4^ and another with concurrent vitamin A deficiency night blindness.^22^

Consequently, with only three reported cases in the literature, we conclude that true cases of vitamin E deficiency in this population are rare; however, further cases may be unreported, and there is controversy over the most appropriate method of diagnosis for vitamin E deficiency,^18^ with experts suggesting vitamin E should be interpreted in the context of serum lipid levels.^18^ Vitamin E levels may be underreported in cases in which there are high circulating lipid levels^35^ and overreported when lipid levels are low; thus, monitoring of the ratio of vitamin E to cholesterol is recommended, calculated as vitamin E (μmol/L) divided by total cholesterol (mmol/L).^36^ Reference ranges vary for different assays, but authors have suggested a ratio of <2.22 as suggestive of depletion.^35^, ^36^

#### Vitamin K

Vitamin K plays a key role in coagulation, vascular calcification, and bone metabolism.^37^ Depletion has been reported 18 months postoperatively in 69% of patients who have undergone PD.^10^ Coagulation is affected by many factors after PD,^38^ and case reports document coagulopathy when vitamin K depletion is present alongside other micronutrient depletions.^34^ There are many factors that influence coagulation, and vitamin K status in the post‐PD population requires further investigation.

#### B vitamins

Two cases of anemia were attributed to a deficiency in vitamin B_6_ (pyridoxine).^39^ Both patients underwent PD and developed anemia within 2 years of surgery. Of note was the concurrent presence of low serum levels of zinc in one patient and zinc and copper in the second. These case reports do not comment on the use of PERT in these patients; one patient gained weight during their recovery, and the second lost >10% of their body weight before stabilizing.

Two cases of biotin deficiency are reported, but these occurred alongside multiple deficiencies in one case^40^ and alongside zinc deficiency, anemia, and malnutrition in a second.^41^ As dietary sources of biotin in food are largely protein based,^40^ it is possible that the concurrent protein deficiency contributed to the biotin deficiency.

Tryptophan deficiency, which leads to niacin deficiencies, is reported in serotonin‐producing NETs, with patients developing pellagra symptoms (diarrhea, dermatitis, and dementia), which respond to niacin supplements.^42^ Niacin status should be considered in those patients who have undergone PD for serotonin‐producing NETs, especially if there is disease recurrence.

Multiple cases of thiamin deficiency are reported, and eight cases of Wernicke's encephalopathy are described—but in each case, there was a contributory factor, such as the use of PN that does not contain thiamin.^43^ A single case of beriberi was reported in a patient 8 years after PD with concurrent diuretic use.^44^ This patient presented with edema, gait abnormalities, and foot numbness.^44^

Vitamin B_12_ deficiency was linked to a case of spinal cord degeneration in a patient 12 months after PD, but this case was complicated by the presence of a copper deficiency.^45^ On occasion, more extensive gastric resection may be carried out alongside PD, and clinicians should be aware of the risk of vitamin B_12_ deficiency in this setting because of the lack of intrinsic factor.^46^

B vitamins are water soluble and not stored in the body; therefore, a regular intake is required,^18^ and serum levels fluctuate considerably. Thus, it is not possible to determine the true incidence of depletion or deficiency, although cases are most likely to occur alongside a more generalized malnutrition, in which vitamin B supplementation is recommended as part of the prevention of refeeding syndrome, for example.^47^ European Society for Clinical Nutrition and Metabolism (ESPEN) guidelines recommend the monitoring of thiamin after bariatric surgery,^18^ which has very similar physiological consequences to PD; but with the exception of folate and vitamin B_12_, B vitamins are not routinely measured in the UK.

### Trace element deficiencies

#### Iron deficiency and anemia

Early signs of iron deficiency are detectable in routine blood tests as low levels of ferritin, transferrin saturation, or iron, although iron levels can fluctuate and blood test results in patients before and after PD require interpretation because altered liver function and inflammation can both falsely elevate markers of iron stores. Inflammation also reduces iron absorption.^48^

Both heme iron (from animal sources) and non‐heme iron (from plant sources) are absorbed in the enterocytes in the duodenum and proximal jejunum.^48^ Very little iron is absorbed from the diet (approximately 1.2–2 mg/day),^48^ and therefore, recycling of iron from the phagocytosis of old red blood cells is crucial for maintaining iron homeostasis.^49^ New tissue growth increases the body's requirement for iron and absorption increases in this setting.^48^

Iron uptake is, in part, regulated by hepcidin, which is synthesized and secreted by the liver. This peptide reduces iron losses into extracellular fluids, increasing cellular iron retention. Hepcidin secretion is stimulated when iron stores are high, but hepcidin levels are increased up to 100 times in inflammatory states, thus resulting in anemia due to inflammation.^50^, ^51^ This is important in patients before undergoing PD because the most common presenting complaint is jaundice, and whereas hepcidin has been shown to be downregulated in patients with cirrhosis due to cholestasis (primary biliary cirrhosis),^52^ upregulation of hepcidin has been shown in animal models with cholangitis.^53^ Observational studies in patients with PDAC have highlighted altered levels of iron regulators, suggesting increased iron use by the cancer cells.^54^ These factors warrant further exploration and are outside of the scope of this paper but highlight the need to assess iron status in patients before and after PD.

Anemia is identified as undertreated after PD, with ongoing depletion of hemoglobin and mean corpuscular volume levels, with all patients either untreated or not responding to treatment with iron and vitamin B_12_.^55^ Although biochemical iron depletion is common, there are limited reports on the incidence of iron deficiency anemia, which occurred in 21%–44% of patients after PD^4^, ^56^ and is recognized as a long‐term complication of PD.^56^ Thus, regular clinical and biochemical assessment is warranted.

#### Calcium

There are limited data exploring calcium depletion specifically, but issues with calcium uptake were initially proposed in 1958 when Nakayama^57^ published experimental data in dogs and one human after TP. He witnessed tetany in a human patient 8 months after TP that resolved with calcium supplementation.^57^

Bone density was not assessed, but parathyroid hormone (PTH) was elevated in 11% of patients after PD in a large cohort study assessing micronutrient status over 10 years.^4^ PTH increases in response to low serum calcium levels and could be considered a marker of poor calcium status. However, low calcium levels in the blood could occur as a result of vitamin D deficiency, renal failure, or calcium malabsorption, and therefore, results should be interpreted alongside serum phosphate, renal function, vitamin D, and magnesium levels.^58^ However, in the healthy older population, there is no relationship between PTH and bone loss.^59^

Because serum calcium levels are maintained by bone resorption,^57^ bone loss and frequency of low‐impact fracture as signs of calcium depletion were studied, as serum calcium level is not a reliable marker of calcium depletion. However, bone loss is limited as a marker of calcium depletion because calcium is not singularly responsible for bone loss, with vitamin D and magnesium playing key roles.^60^, ^61^

Bone loss has been studied in a large propensity‐matched study of 8080 patients and demonstrated that those who had undergone major pancreatic resection had a 2.4‐fold increase in pathological fractures (*P* < 0.0001) and increased risk of osteopenia and osteoporosis (*P* < 0.0001).^30^ Of all the resections studied, PD was associated with an increased rate of bone mineral loss (*P* < 0.05),^30^ consistent with other studies.^62^

Studies report the incidence of osteopenia at 49%^8^–61%^63^ and osteoporosis at 29%,^8^ although the variable time frames of postsurgery assessment should be acknowledged.

Interestingly, some authors have suggested that loss of bone density may be part of the cachexia syndrome and have identified a relationship between bone density and survival. This is outside of the scope of this paper, but it may have an impact on the interpretation of bone density in those with active malignancy.^63^, ^64^

In summary, although impaired calcium uptake is implied, this cannot reliably be assessed, and bone density assessment as a marker of overall bone health should be carried out routinely. Bone density assessment is recommended every 2–5 years in other cohorts at risk of malabsorption,^65^, ^66^ and in the absence of any data, it seems pragmatic to adopt this approach.

#### Copper

Studies exploring symptoms in copper deficiency report largely neurological presentations, cardiac abnormalities, poor wound healing, neutropenia, osteoporosis, limb weakness, hair depigmentation, and anemia,^18^, ^67^, ^68^, ^69^ and biochemical depletion has been reported in patients receiving jejunal enteral nutrition^70^ and in those undergoing bariatric surgery,^71^ further supporting the impact of the reduction in the proximal small bowel absorptive capacity. However, a literature search revealed only one case after PD, complicated by a vitamin B_12_ deficiency. In this case, the patient had a 10‐year history of using denture creams containing high amounts of zinc and had a high serum zinc level, which may have been the cause of copper depletion.^45^ Clinicians should be aware that copper uptake can be inhibited with high zinc provision.^72^

Therefore, it appears that copper deficiency is rare but, again, has the potential to be underreported. ESPEN guidelines recommend copper levels should be measured after surgeries that remove or bypass the duodenum and in patients receiving jejunal feeding.^18^

#### Magnesium

On a cellular level, magnesium is closely related to potassium,^73^ and deficiency symptoms range from secondary depletion of other electrolytes to ataxia, seizures, cardiac arrythmia, and bone mineral loss.^60^, ^74^, ^75^, ^76^

Although magnesium is not usually considered to be a trace element, depletion is reported after PD, with variable results.^4^, ^11^ Magnesium is labile, and increased losses are seen with diarrhea,^73^ excess alcohol^77^ and malabsorption.^73^ Patients after PD are often treated with proton pump inhibitors, and there is a high risk of diabetes, for which drugs such as metformin may be used. Both of these medications may contribute to hypomagnesemia.^74^, ^75^

Thus, although magnesium depletion occurs after PD, it is likely to be multifactorial and warrants surveillance.

#### Selenium

No cases of symptomatic selenium deficiency in patients after PD were identified. However, symptoms can be extrapolated from a case series of four Japanese patients receiving long‐term enteral nutrition (with a non–selenium‐containing formula), two of whom had undergone PD and a third a gastro‐pancreatectomy.^78^ Patients presented with muscular pain, gait disturbances, and leg weakness, but symptoms resolved after a 10–20‐day course of intravenous selenium supplements. Thiamin levels were also low in this cohort, and these patients had been fed enterally for between 7 and 11 years. These cases are not consistent with current management in Europe, as all enteral formulations across Europe are nutritionally complete for micronutrients.^47^

In summary, no cases of selenium deficiency were identified in the published literature related to PD that were not attributed to the provision of inadequate selenium. However, these cases demonstrated deficiency symptoms that were only apparent after 7–11 years of inadequate intake. Because depletion is reported^5^, ^6^ and deficiency has a significant morbidity, high‐risk patients (proposed as those not taking PERT or those with concurrent malnutrition) should be screened for selenium depletion.

#### Zinc

Zinc is absorbed in both the duodenum and jejunum.^48^ Excess zinc is excreted initially in bile and reabsorbed via the enterohepatic circulation^18^; thus, there may be increased losses in those undergoing external biliary drainage, which may be used before PD in patients presenting with obstructive jaundice.^79^ Both intestinal and urinary losses are reduced during periods of deficiency, with studies suggesting humans can regulate their zinc absorption and excretion with a 10‐fold change in dietary intake.^80^ Zinc status plays a key role in the immune system,^81^ and there are some data that suggest that the exocrine pancreas cells secrete a ligand that enhances absorption of zinc in the jejunum^80^; thus, zinc uptake could be impaired in patients with PEI, although studies suggest up to 2 mg of zinc could be lost per day in pancreatic secretions,^48^ which will be reduced after PD because there is less pancreatic tissue. Zinc uptake might be impaired in patients who consume diets high in phytates (found in legumes, nuts, and grains)^82^; thus, extra care should be taken in those following more plant‐based diets. Furthermore, muscle is the largest store of zinc in the body, holding 57% of zinc stores,^80^ so depleted muscle mass may further reduce zinc status. Plasma zinc is readily available but can fluctuate throughout the day and should be interpreted alongside serum albumin and CRP levels.^18^

There is a physiological link between iron and zinc, with iron uptake increased when zinc levels are high within the gut and iron uptake impaired with zinc deficiency.^48^ Zinc and copper are also closely related, with zinc depletion enhancing copper absorption and zinc supplementation inhibiting uptake of copper from food.^72^

Zinc deficiency presents with a variety of clinical manifestations including glossitis, alopecia, nail dystrophy, poor wound healing, reduced immune function, photophobia, diarrhea, hypogeusia, and a distinct skin manifestation referred to as acquired acrodermatitis enteropathica (AE).^18^, ^83^ Biochemical deficiencies are reported in between 4% and 83% of cases,^4^, ^5^, ^8^, ^9^, ^11^ with a small study reporting dietary deficiency in 50% of cases.^3^

A retrospective review of 206 patients with PDAC who underwent surgery (including PD, TP, and distal pancreatectomy) identified 36 (17.5%) patients with zinc depletion.^84^ On multivariant analysis, the zinc‐deficient group had a higher incidence of infectious complications (61.1% vs 31.8%, *P* = 0.002) and a higher number of surgical complications defined as a Clavien‐Dindo score of ≥3, although this did not meet significance (50% vs 34.1%, *P* = 0.088). There was no difference in clinically relevant pancreatic fistula, delayed gastric emptying, or thrombotic complications.^84^ There was no difference in comorbidities, body mass index, or nutrition risk scores (using the Controlling Nutritional Status [CONUT] score) between the zinc‐deficient and zinc‐sufficient group.^84^

A large prospective study assessed serum zinc levels in 329 patients who had undergone pancreatic surgery (52% PD) between 0 and 203 months previously (median, 41 months) and identified zinc depletion in 70% of cases.^85^ The authors attempted to look for correlations and, on multivariate analysis, identified zinc depletion was more common in men (odds ratio [OR], 3.7; *P* = 0.001), those with serum albumin level of <3.9 g/dl (OR, 6.36; *P* < 0.001), aspartate aminotransferase levels >51 U/L (OR, 4.6; *P* < 0.001), or those who had undergone TP (OR, 3.68; *P* = 0.009).^85^ Although the authors discussed the potential for a correlation with PEI, no data were recorded on the presence or treatment of PEI.

An earlier study carried out in Taiwan identified not only a correlation with PEI but also a resolution of symptoms of zinc deficiency after 4 weeks or treatment with PERT in 33 patients with zinc deficiency after PD from a total cohort of 48.^86^ The authors identified skin rash, photophobia, and glossitis as the most common symptoms in those with zinc deficiency and, importantly, identified a difference in stool fat losses between the two groups (*P* = 0.038) but no difference in fecal elastase (*P* = 0.160), further supporting the concern that fecal elastase is not accurate after PD.^86^

Resection of the duodenum is only one of several mechanism suggested to explain the occurrence of zinc deficiency. Zinc is transported in the body predominantly by albumin and other amino acids; thus, a role for protein malabsorption has been proposed.^87^, ^88^ Indeed, as zinc‐rich foods are typically protein‐rich foods, poor protein intake is likely a contributory factor.^83^ Zinc deficiency has also been widely associated with diarrheal based diseases,^83^ although it is not possible to differentiate zinc deficiency as a causal factor or an effect of diarrhea, as the cases are predominantly accompanied by significant malnutrition.^83^

Essential fatty acid (EFA) deficiency presents with similar clinical manifestations to zinc deficiency.^89^ One study reported EFA deficiency occurring alongside zinc deficiency, proposing that AE could occur as a result of combined nutrient deficiencies.^11^ If this is the case, this will make a determination of the true incidence of deficiency difficult to ascertain because in many developed countries, nutrition intervention is routine and may prevent the significant malnutrition often required to see a manifestation of zinc deficiency.

A literature search revealed eight cases of clinical manifestations of zinc deficiency after PD. In all of these cases, zinc deficiency is reported alongside multiple deficiencies and protein‐energy malnutrition (Table 2).

**Table 2:** Description of cases of zinc deficiency occurring after PD.

Source	Country	Duration after PD	Nutrition status	Other micronutrient depletion	Signs and symptoms	Treatment
Jang et al^91^	Korea	7 years	25‐kg weight loss	Energy	Necrolytic migratory erythema and stomatitis	Parenteral nutrition; PERT
Kim et al^87^	Korea	6 years	10‐kg weight loss; BMI, 15 kg/m^2^; no PERT	Vitamin A, cholesterol, transferrin, calcium, magnesium, anemia	Refractory dermatitis, alopecia, pitting edema, loose stool, erosion of fingertips, glossitis, new‐onset diabetes	Parenteral nutrition for 1 month; maintained on albumin and lipid infusions and oral zinc
Yu et al^90^	Taiwan	15 months	Not specified	Anemia	Acrodermatitis enteropathica, alopecia, glossitis, nail dystrophy	Intravenous zinc, PERT, and parenteral amino acids
Yu et al^90^	Taiwan	4 years and 13 years	Weight stable	Hypoalbuminemia	Itchy erythematous plaques with scales, hyperpigmentation, and tightly shining skin; amenorrhea and alopecia; ulceration on toes	Oral zinc sulphate, parenteral nutrition, and PERT
Yazbeck et al^41^	Beirut	3 years	4‐month history of vomiting undigested food, 4‐kg weight loss	Anemia; low levels of biotin, vitamin D, and zinc	Alopecia, total body hair loss, dry skin with scales, maculopathy with significant visual loss, glossitis, amenorrhea, fatty liver	Intravenous zinc, enteral nutrition, and PERT; maintained on PERT and multivitamins
Kanagalingam et al^93^	USA	5 years	Low BMI	Anemia, magnesium	Desquamating rashes, bilateral edema in the lower extremities; skin biopsies showing mild psoriasiform dermatitis with confluent parakeratosis, suggestive of a nutrition cause	220 mg of oral zinc alongside intravenous iron
Hata et al^92^	Japan	69 days	Not stated, but protracted admission	Hypoalbuminemia, low copper	Acrodermatitis enteropathica, taste changes, glossitis	Parenteral nutrition, PERT and intravenous followed by oral zinc

Abbreviations: BMI, body mass index; PD, pancreatico‐duodenectomy; PERT, pancreatic enzyme replacement therapy.

In seven of these eight cases, zinc deficiency presented >12 months after PD and alongside malnutrition. In most cases, patients were not taking PERT,^41^, ^87^, ^90^, ^91^ and in four cases, PERT was part of the management,^87^, ^90^, ^91^ suggesting malabsorption may play a role. In the case presented by Hata et al, the potential long‐term recurrent nature of this deficiency was highlighted, but insufficient information was available to assess contributory factors such as dietary intake, adherence to PERT, concurrent malnutrition, or nutrition status before surgery.^92^ Nutrition support in the form of either PN or enteral nutrition was used in all cases.^41^, ^87^, ^90^, ^91^, ^93^

Zinc depletion occurred in two studies despite supplementation, with one study reporting an incidence of zinc depletion of 46% in patients taking a multivitamin and mineral containing 15 mg of zinc^4^ and 17.8% zinc depletion in those supplemented with 50 mg of zinc.^94^

From the literature, it is not possible to distinguish between singular causes of the clinical manifestations, and therefore, nutrition assessment for zinc deficiency should be part of a more comprehensive review. The increased risk of infectious complications and significant surgical complications suggests early identification and correction of zinc deficiency may reduce surgical morbidity.^84^

## TREATMENT OF DEFICIENCIES

The ESPEN micronutrient guidelines recommend that supplementation should be carried out via the oral or enteral route if this is safe and effective but acknowledge there are no clinical trial data.^18^ Nutrition recommendations for minimal levels of nutrient intake to prevent depletion vary between countries.^15^, ^95^ Corrective doses are higher—and where recommendations exist, they are summarized in Table 3—but oral doses in those with deficiency due to poor oral intake are based on normal gastrointestinal absorption. Thus, it is likely that with impaired uptake, corrective doses may need to be higher or given intravenously.

**Table 3:** Treatment of micronutrient deficiencies after pancreatico‐duodenectomy.

Micronutrient role in the body and recommended daily intake	Signs and symptoms of deficiency and cautions for interpreting results	Treatment	When to recheck and maintenance therapy
**Vitamin A** Development of visual pigments in rods and cones of the eye, maintains conjunctiva, skin integrity, immune function, embryonic development^18^, ^22^, ^95^ DRI: 700–900 μg/day^96^ RNI: 600–700 μg/day^97^	Night blindness, xerophthalmia, anemia, bitot spots; can lead to permanent blindness^22^, ^95^ CAUTION: Blood samples should be protected from the light and processed rapidly. CAUTION: Serum levels are affected by inflammation.	Optimize PERT. Ophthalmology assessment Ensure patient is not following an unnecessary low‐fat diet. Mild deficiency can be treated with oral dosing of 3300–350 IU vitamin A/retinol or 990–1050 μg retinol equivalent.^18^, ^21^ Where absorption is impaired and oral supplementation is ineffective or night blindness is present, provide intravenous supplementation at a dose of parenteral daily supplements (800–100 μg retinol equivalent) 2–3 times per week for a month in addition to high‐dose oral supplementation.^a^	Repeat ophthalmological assessments at 2 months to ensure improvements. Recheck serum levels every 3 months until normalized. Consider long‐term daily oral supplementation using a multivitamin and mineral capsule. Recheck annually.
**Vitamin D** Bone metabolism, calcium homeostasis DRI: 20 μg/day^96^ RNI: 10 μg/day^97^	Fatigue, loss of bone density, elevated PTH CAUTION: Renal failure—vitamin D_3_ is hydroxylated to 25‐hydroxyvitamin D_3_ in the liver and then to 1,25‐dihydroxyvitamin D_3_ in the kidneys.^98^ Thus, 1,25‐hydroxyvitamin D_3_ must be assessed and supplemented in those with renal failure.	Optimize PERT. Provide bone health advice; include sunlight exposure, weight‐bearing exercise, and smoking and alcohol cessation. Initial treatment: up to 125 μg or 5000 IU daily orally^18^ If unsuccessful or severely deficient, loading doses may be given weekly for up to 6 weeks. Intramuscular doses are available^18^ but not normally needed.	DEXA scan every 2–5 years Recheck serum vitamin D levels every 3 months until normalized. Titrate daily oral supplementation to maintain normal vitamin D and PTH levels. Ensure high doses are reviewed regularly because of the risk of toxicity.
**Vitamin E** Preventing membrane oxidation, protective against oxidative damage^18^, ^99^ DRI: 15 mg/day^96^ RNI: 3–4 mg/day^15^	Myopathy, polyneuropathy, peripheral neuropathy, balance and coordination disorders CAUTION: Blood samples should be protected from the light and processed rapidly.	Optimize PERT. Ensure patient is not following an unnecessary low‐fat diet. 100–200 mg/day for 3 months for depletion^18^ Consider intravenous supplementation if patient is symptomatic.	Recheck with lipid levels (ratio to cholesterol levels); aim for ratio >2.2. Reevaluate every 3 months; supplementation might be needed long‐term. Consider long‐term daily oral supplementation using a multivitamin and mineral capsule.
**Vitamin K** Coagulation, vascular calcification, and bone metabolism^37^ DRI: 90–120 g/day^96^ RNI: 1 μg/kg/day^15^	Coagulopathy, loss of bone density CAUTION: Patients on anticoagulation or with concurrent liver disease. Assessment of clotting should be undertaken with supplementation.	Optimize PERT. No data are available for dose recommendation.	No data are available to support recommendations; long‐term surveillance of coagulation markers is suggested.
**B vitamins** Diverse function as coenzymes in metabolism, immunity, and preservation of neurological function^18^ Thiamin o DRI: 1.1–1.2 mg/day^96^ o RNI: 0.8–0.9 mg/day^97^ Folic acid o DRI: 400 μg/day folate^96^ o RNI: 200 μg/day folate^97^ Vitamin B_12_ o DRI: 2.4 μg/day^96^ o RNI: 1.5 μg/day^97^ Riboflavin o DRI: 1.1–1.3 mg/day^96^ o RNI: 1.1–1.3 mg/day^97^ Niacin o DRI: 11–16 mg/day^96^ o RNI: 12–16 mg/day^97^ Pantothenic acid o DRI: 5 mg/day^96^ o Safe: 3–7 mg/day^15^ Pyridoxine o DRI: 1.5–1.7 mg/day^96^ o RNI: 1.2–1.4 mg/day^97^ Biotin o DRI: 30 μg/day^96^ o Safe: 10–200 μg/day^15^	Thiamin: apathy, short‐term memory, confusion, Wernicke‐Korsakoff encephalopathy, beriberi (edema, gait abnormalities, congestive heart failure) Folate/folic acid: megaloblastic anemia, glossitis, pancytopenia, depression, psychosis, anorexia, fatigue Vitamin B_12_: anemia, thrombocytopenia, peripheral neuropathy, vertigo, irritability, psychosis, dementia, glossitis, fatigue Riboflavin: glossitis; angular stomatitis; itchy eyes; photophobia; normocytic anemia; seborrheic dermatitis of the face, trunk, and scrotum Niacin: pellagra (diarrhea, dermatitis, dementia) Pantothenic acid: postural hypotension, tachycardia, numbness/burning of hands, extreme fatigue, irritability (usually occurs with severe malnutrition) Pyridoxine: glossitis, microcytic anemia, confusion, convulsions, angular stomatitis, depression Biotin: dermatitis, alopecia, ataxia	Provide nutrition support if indicated, with particular focus on protein intake. B vitamin supplementation should be commenced immediately in those at risk of refeeding syndrome.^47^ Thiamin: high‐dose thiamin in those with encephalopathy (200–500 mg/day for at least 10 days)^18^ Folic acid: 1–5 mg/day^18^ Vitamin B_12_: 350 mg orally daily, or 1000–2000 μg every 1–3 months^18^ Riboflavin: 5–10 mg/day orally. 160 mg IV for 4 days can be used if severe deficiency^18^ Niacin: doses in excess of 40 mg/day Pantothenic acid: combination high‐dose B vitamins Pyridoxine: 50–100 mg for 1–2 weeks orally Biotin: 10 mg/day orally	B vitamin supplementation in this cohort is unusual and likely to be combined with nutrition support. Once adequate dietary, enteral, or parenteral nutrition is established, diet should contain adequate doses for maintenance. Thiamin should be supplemented in line with refeeding/alcohol excess guidelines when necessary.^18^ Vitamin B_12_ and folic acid doses should be titrated to serum levels. In the absence of adequate biochemical testing in most centers, consider long‐term daily oral supplementation of combination of B vitamins within a multivitamin and mineral capsule in those with poor nutrition status. Local guidelines should be followed for those with ongoing high alcohol intakes.
**Iron** Required for energy and substrate metabolism DRI: 8 mg/day (18 mg/day in women aged 19–50 years)^96^ RNI: 8.7 mg/day (14.8 mg/day in women aged 11–50 years)^97^	Biochemical: low ferritin or transferrin saturation with or without anemia (low hemoglobin/hematocrit) Symptoms: feeling cold, short of breath on exertion, postural dizziness CAUTION in interpreting results with liver disease, cholangitis, and inflammation—results can be elevated	Initial treatment: oral iron for 3 months (100–200 mg/day iron in divided doses, taken daily or every other day where poorly tolerated^18^) In the event of ineffective treatment, or the need for a rapid response (before surgery), consider intravenous supplementation (be aware of the risk of skin staining/anaphylaxis). High dietary sources of iron should be recommended alongside supplementation but are unlikely to be effective in isolation.	Recheck iron studies after 3 months. There were no data on maintenance doses, but data support recurrent cases.^4^ Encourage high dietary sources of iron and PERT. Consider oral iron taken 2–4 times per week in those unable to maintain iron stores without supplementation.^a^ Recheck 6‐monthly once normalized.^a^
**Calcium** Required for cellular structure and metabolic functions; structural role in bones and teeth^15^ DRI: 1000 mg/day^96^ RNI: 700 mg/day^97^	Moderate deficiency: elevated PTH, bone density loss Severe deficiency: tetany	Ensure adequate oral dietary calcium uptake and absorption using PERT. 1000 mg of calcium and vitamin D supplements should be provided routinely. Severe cases require inpatient admission and cardiac monitoring while intravenous calcium supplements are administered.^a^ Measure serum calcium levels daily until levels are normalized in severe cases.	Ensure high dietary sources of calcium and PERT. 800–1000 mg calcium (with vitamin D) supplements Long‐term assessment uses secondary assessment methods. o Vitamin D and PTH every 6–12 months o Bone density scans every 2–5 years CAUTION: Long‐term supplementation in those with high dietary intake/good absorption may cause kidney stones^96^—in patients at risk, consider singular vitamin D supplement.
**Copper** Regulation of growth, metabolism, connective tissue maturation, neurotransmission, melanin production, immune function, thyroid hormone, and glucose regulation^18^ DRI: 0.9 mg/day^96^ RNI: 1.2 mg/day^97^	Acute symptoms: cardiac arrhythmias, myeloneuropathy, poor wound healing Chronic symptoms: microcytic anemia, neutropenia, osteoporosis, hair depigmentation^18^, ^67^, ^68^ CAUTION: Copper update is impaired with zinc supplementation. Copper levels should be checked when long‐term zinc supplementation is required.	In moderate deficiency (defined as 8–11 μmol/L or 50–70 μg/ml with normal CRP and ceruloplasmin levels): 2–5 mg/day copper gluconate orally^18^ In severe cases (defined as <8 μmol/L or 50 μg/ml or defined as 8–1 μmol/L or 50–70 μg/ml with high CRP or low ceruloplasmin levels): 4–8 mg/day intravenously via a central line (risk of phlebitis with infusion of >2 mg/day)^18^	No data are available on duration of treatment or frequency of assessment. In those with acute symptoms: o Consider administering treatment daily until clinical signs of recovery are observed or for at least 2 weeks. o Clinical experience suggests repeated measurement of serum levels weekly to observe a pattern. Once serum levels normalize, continue with oral supplementation until clinical symptoms resolve. Maintenance therapy: A maintenance dose of double the RNI, with reassessment every 3 months until stable and every 6–12 months after that, is suggested.
**Magnesium** Skeletal development, cofactor for enzymes, RNA synthesis, and replication of DNA^15^ DRI: 265–300 mg/day^96^ RNI: 270–300 mg/day^97^	Low serum levels cause ataxia, seizures, arrythmia, bone loss,^60^, ^74^, ^75^, ^76^ and secondary hypokalemia. NOTE: Patients are at increased risk with ongoing diarrhea, alcohol excess, PPI, and metformin use.^73^, ^77^	Severe deficiency requires inpatient admission and cardiac monitoring while magnesium is corrected intravenously. Maintenance doses should be titrated to serum levels.	Daily assessment initially during correction in severe deficiency; otherwise, every 2–4 weeks until levels normalize and then every 3 months for the first year Titrate daily oral supplementation to maintain normal magnesium. Annual assessment if level remains stable or every 6 months if patient is taking maintenance magnesium supplements
**Selenium** Required for the synthesis of selenoproteins, antioxidant role, control of thyroid metabolism^18^ DRI: 55 μg/day^96^ RNI: 60–75 μg/day^97^	Weakness, cardiomyopathy, skin disorders, rheumatic arthritis^100^ CAUTION: CRP level >10 mg/dl can reduce plasma selenium levels^101^	Provide nutrition support if indicated. Assess for undertreated PEI and optimize PERT if necessary. Most deficiencies can be corrected with 200 μg/day orally.^22^ If patient has poor gastrointestinal function or serum levels, <0.4 μmol/L at 100 μg/day can be given intravenously for 7–10 days. Higher doses may be required.^18^	Ensure adequate intake (most cases occur in unsupplemented artificial nutrition). Depletion: o Recheck every 3 months in patients with biochemical depletion to ensure adequacy of treatment. o Recheck every 6 months for 1 year and then annually. Deficiency: o Recheck levels after 1 month of treatment to ensure upward trend. o Consider long‐term daily oral supplementation using a multivitamin and mineral capsule. o Review at 3 months; if normalized, stop high‐dose supplements and recheck every 6 months. o Recheck every 6 months for 1 year and then annually.
**Zinc** Hundreds of functions within structural, catalytic, and regulation pathways; plays a key role in growth and wound healing^18^ DRI: 8–11 mg^96^ RNI: 7–9.5 mg/day^97^	Glossitis, alopecia, nail dystrophy, poor wound healing, reduced immune function, photophobia, diarrhea, hypogeusia, acquired acrodermatitis enteropathica^18^, ^83^ NOTE: hypothetical increased risk in those with low muscle mass due to depleted stores NOTE: increased risk in those with ongoing biliary drainage due to zinc reabsorption via the enterohepatic circulation CAUTION: Results should be interpreted with caution in inflammation.	Provide nutrition support if indicated. Assess for undertreated PEI and optimize PERT if necessary. 0.5–1 mg/kg/day elemental zinc can be given orally for 3 month.^18^ Zinc sulfate and zinc chloride may not be as well tolerated as zinc histidinate, zinc gluconate, or zinc orotate.^18^	Ensure diets very high in phytates (legumes, nuts, and grains) are avoided.^82^ Check iron and copper levels when supplementing zinc to assess for secondary depletion. Depletion: Recheck every 3 months in patients with biochemical depletion to ensure adequacy of treatment. Deficiency: o Monthly assessment is advised in patients with clinical symptoms o In cases of acrodermatitis enteropathica, zinc supplementation should be lifelong at 3 mg/kg/day of elemental dose. This dose may need to be adjusted to achieve normal serum/plasma levels.^18^ o In other cases, a multivitamin and mineral or low‐dose elemental zinc may be required. o Recheck every 6 months for 1 year and then annually.

Abbreviations: CRP, C‐reactive protein; DEXA, dual‐energy x‐ray absorptiometry; DRI, dietary reference intake (United States and Canada) displayed for adults; PEI, pancreatic exocrine insufficiency; PERT, pancreatic enzyme replacement therapy; PPI, proton pump inhibitor; PTH, parathyroid hormone; RNI, reference nutrient intake (United Kingdom) displayed for adults.

^a^ Based on clinical experience.

**Figure 1:**
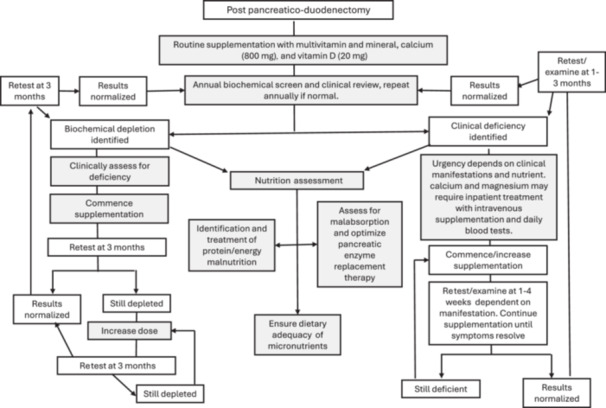
Treatment algorithm for the management of micronutrient depletion/deficiency after pancreatico‐duodenectomy.

Treatment of deficiency can be challenging, and the cases identified in this review all demonstrated different treatment plans. In this clinical setting, it is important to include more generalized nutrition assessment and treatment of malabsorption. In some cases, this must be undertaken quickly because acute deficiency can be life‐threatening (ie, magnesium or calcium). Conversely, iron depletion can take months to resolve. This makes specific treatment recommendations difficult. Essentially, in nonurgent treatment of deficiency or in biochemical depletion, oral therapy should be commenced and reviewed to ensure efficacy. If this is effective, treatment should continue until symptoms resolve and biochemical levels stabilize. If oral therapy does not improve symptoms or biochemical levels, higher‐dose oral or intramuscular/intravenous treatment may be required. Figure 1 proposes a step‐by‐step process for treatment and reevaluation.

Some micronutrient deficiencies may present acutely and require admission or occur alongside other conditions requiring inpatient treatment. In this instance, biochemical testing must be interpreted in the clinical context, acknowledging the impact of inflammation, anemia, or hypoalbuminemia.^19^, ^20^ Where one micronutrient deficiency is present and thought to be associated with malabsorption, clinicians should ensure they assess for other deficiencies.

It is important to remember that deficiencies may occur because of malabsorption after PD, but also because of dietary inadequacy, so a detailed nutrition assessment is an important part of initial evaluation, and dietary adequacy should be encouraged and supported. Where malabsorption is suspected, treatment with PERT should be commenced or dose escalated alongside correction of deficiency and maintained to reduce the risk of the deficiency recurring.

## LIMITATIONS

This review explores multiple micronutrient deficiencies, but data were not available on chromium, cobalt, manganese, iodine, molybdenum, or fluoride. General advice regarding detection and treatment of deficiency of these is available in the ESPEN micronutrient guidelines^18^ but may not reflect treatment with poor gastrointestinal uptake. Data analysis is reliant on clinicians reporting cases of deficiency and the inclusion of these outcomes in studies; thus, is it possible that frequency is underreported, and the presence of multiple deficiencies renders clinical assessment challenging.

Most prescribable oral nutrition supplements contain vitamins and minerals, and some are nutritionally complete,^47^ meaning that the provision of energy and protein supplements may indirectly treat micronutrient depletion because of the presence of micronutrients within these products, thus masking the true frequency.

## CONCLUSION

Micronutrient depletion is common in patients after PD, but true deficiency states appear rare. However, the data are largely based on case studies, with no large‐scale studies identifying true incidence rates. Furthermore, signs and symptoms are diverse and may be associated with other conditions or treated as part of generalized treatment of malnutrition, thus resulting in underreporting.

## RECOMMENDATIONS FOR CLINICAL PRACTICE

The literature is consistent in reporting both iron depletion and anemia as common, and thus, iron deficiency should be regularly screened for. Vitamin D depletion is also common, and in combination with this, reduced calcium uptake, magnesium depletion, and an increased fracture rate supports routine supplementation of calcium and vitamin D in addition to regular bone density assessment.

Zinc depletion is common but clinical manifestations appear to occur alongside severe malnutrition and in geographical regions where PERT supplementation is not routine. However, zinc levels are readily available, and it is possible that depletion is treated before deficiency symptoms are apparent; thus, screening should be routine. Although copper deficiency was rare, the relationship between copper and zinc means both should be analyzed at the same time.

Case reports in vitamins A, E, and K; B vitamins; and selenium are described, but there were multiple factors contributing to these presentations, and most patients had untreated malabsorption and malnutrition.

Because of the costs of micronutrient blood tests, a pragmatic approach should be adopted for the surveillance of status, and we recommend routine assessment (annually) in all patients. This should consist of a clinical review of signs and symptoms and biochemical assessment of iron, zinc, and vitamin D with hemoglobin levels, PTH, and bone density assessment. This will support early diagnosis of clinical manifestations, but in patients with concurrent malnutrition, or where adherence to PERT is poor, a more comprehensive and more frequent assessment is recommended (Table 4).

**Table 4:** Proposal for frequency of micronutrient assessment in patients after pancreatico‐duodenectomy.

	Patients who are nutritionally well and taking PERT	Patients who are malnourished or not taking/not adhering to PERT	Patients with active malignancy despite treatment
Anemia screen: hemoglobin, iron studies, ferritin	Annually	Every 6 months	Every 3–6 months
Bone health: vitamin D and parathyroid hormone	Annually	Every 6 months	N/A
Bone density: dual‐energy x‐ray	Every 3–5 years from 12 months postoperatively	Every 3–5 years from 12 months postoperatively	N/A
Zinc and copper	Annually	Annually	N/A
Markers of inflammation/assessment of confounding variables: CRP level, liver function tests, serum albumin level	Annually	Every 6 months	Every 3 months
Other trace elements: selenium, magnesium	Not required unless clinical signs of deficiency suspected	Annually	Test if clinical symptoms
Other fat‐soluble vitamins: vitamins A and E	Not required unless clinical signs of deficiency suspected	Annually	Test if clinical symptoms
Coagulation: vitamin K/coagulation panel	Not required unless clinical signs of deficiency suspected	Annually	Not required unless clinical signs of deficiency suspected
B vitamins	Not required unless clinical signs of deficiency suspected	Not required unless clinical signs of deficiency suspected	Not required unless clinical signs of deficiency suspected

Abbreviations: CRP, C‐reactive protein; N/A, not applicable; PERT, pancreatic enzyme replacement therapy.

Although there are no data in patients with active malignancy, a pragmatic approach should be adopted to consider routine assessment of only the micronutrients with the potential to have an immediate impact on quality of life. In this case, we recommend biochemical screening for anemia and other testing only if clinical deficiency is suspected (Table 4).

Further work should focus on identifying the true incidence of deficiency and optimizing therapy to ensure the impact of deficiency is prevented by routine screening and prompt treatment of depletion.

## AUTHOR CONTRIBUTIONS

**Mary E. Phillips**: Conceptualization; investigation; methodology; visualization; writing—original draft; writing—review and editing. **Callum Livingstone**: Supervision; validation; writing—review and editing. **Adam E. Frampton**: Supervision; writing—review and editing. **Kathryn H. Hart**: Conceptualization; supervision; validation; writing—review and editing.

## CONFLICT OF INTEREST STATEMENT

Mary E. Phillips has received honoraria for teaching and expert panel attendance from Viatris, Nutricia Clinical Care, and PharmaNord. The remaining authors declare no conflicts of interest.
